# One to Two Cycles of Consolidation Chemotherapy With Capecitabine After Neoadjuvant Chemoradiotherapy Does Not Benefit Low-Risk Patients With Locally Advanced Middle-Low Rectal Cancer

**DOI:** 10.3389/fonc.2021.695726

**Published:** 2021-09-30

**Authors:** Xueqing Sheng, Shuai Li, Yangzi Zhang, Jianhao Geng, Hongzhi Wang, Xianggao Zhu, Jizhong Quan, Yongheng Li, Yong Cai, Weihu Wang

**Affiliations:** ^1^ Key Laboratory of Carcinogenesis and Translational Research (Ministry of Education/Beijing), Department of Radiation Oncology, Peking University Cancer Hospital & Institute, Beijing, China; ^2^ Department of Radiation Oncology, Jilin Guowen Hospital, Gongzhuling, China

**Keywords:** neoadjuvant chemoradiotherapy, capecitabine, consolidation chemotherapy, low-risk rectal cancer, complete response

## Abstract

**Background and Objective:**

Organ preservation can enable locally advanced rectal cancer (LARC) patients with clinical complete response (cCR) after neoadjuvant treatment to maintain quality of life. In this study, we aimed to evaluate whether one or two cycles of capecitabine after neoadjuvant chemoradiotherapy (NCRT) without extending the interval between the end of NCRT and surgery could increase the complete response (CR) rate in low-risk middle-low LARC patients.

**Material and Methods:**

We retrospectively evaluated middle-low LARC patients with low risk defined as clinical T2-3b, mesorectal fascia-clear, and extramural vascular invasion-negative by magnetic resonance imaging (MRI), treated between January 2015 and July 2019. Patients were divided into two groups according to whether consolidation chemotherapy was administered after NCRT. Patients in the consolidation chemotherapy group received one or two cycles of capecitabine (1000 mg/m^2^ twice daily from days 1 to 14). The main outcome was the CR rate, including pathological CR (pCR) and cCR.

**Results:**

A total of 169 patients, 105 in the consolidation chemotherapy group and 64 in the non-consolidation chemotherapy group, were included in the study, and the median follow-up was 37.2 months (range, 0.4–71.2 months). Seventeen patients achieved cCR and the remaining 152 underwent surgery after neoadjuvant treatment. There was no significant difference in the CR rate (39.0% *vs*. 35.9%, p=0.686), ypT0-2N0 rate (65.2% *vs*. 63.3%, p=0.812), or ypN0 rate (83.7% *vs*. 88.3%, p=0.503) between the consolidation chemotherapy and non-consolidation chemotherapy groups. Among the patients achieved cCR, 3 (17.6%) experienced regrowth in the rectum and 2 (11.8%) experienced distant metastasis. There was also no significant difference in the 3-year disease-free survival (87.4% *vs* 85.9%, p=0.971) in patients who underwent surgery between the two groups. Multivariate logistic regression analysis indicated that normal Carcinoma Embryonic Antigen (CEA) levels (p = 0.001) were associated with a higher CR rate. Moreover, there were no significant differences in the incidences of grade ≥2 acute toxicities during neoadjuvant treatment.

**Conclusion:**

Although there was no increase in treatment-related toxicities between the two groups, simply adding one or two cycles of capecitabine after NCRT might be insufficient to benefit low-risk middle-low LARC patients.

## Introduction

Colorectal cancer (CRC) is the third most frequently diagnosed cancer and the second leading cause of cancer-related deaths worldwide (1). Because its symptoms are not obvious, most rectal cancers are locally advanced or advanced at the initial diagnosis. Neoadjuvant chemoradiotherapy (NCRT) followed by total mesorectal excision (TME) is the standard treatment for locally advanced rectal cancer (LARC) (2). However, the response differs in patients receiving NCRT. Approximately 50% to 60% of patients are down-staged after NCRT, and approximately 20% have a pathologic complete response (pCR) (3–7). Patients with pCR and ypT1-2N0 have a better long-term prognosis (3, 4).

To ensure a curative effect of surgery after NCRT, the anus is removed in some patients with middle and low rectal cancer, which has a profound impact on the subsequent quality of life. Therefore, non-surgical treatment strategies, so-called “watch-and-wait” approaches, can be considered for patients with clinical complete response (cCR) after NCRT. Habr-Gama et al. first reported the long-term results of rectal cancer patients who underwent this approach after NCRT, and these patients had excellent survival outcomes and better bowel function than patients who underwent surgery (8). Many related studies were then performed (9–11) and the International Watch and Wait Database was established, which bring benefits to patients who desire to preserve the anus or cannot tolerate surgery.

To improve the complete response (CR) rate, strategies such as adding induction or consolidation chemotherapy before surgery, referred to as total neoadjuvant therapy (TNT), have been proposed. One retrospective study at the Memorial Sloan Kettering Cancer Center indicated that adding induction chemotherapy could lead to higher pCR rates (12). The CAO/ARO/AIO-12 study showed that consolidation chemotherapy with extended interval between NCRT and surgery led to a higher CR rate than induction chemotherapy (13). Data from the National Cancer Database (NCDB) indicate that an extended interval time alone between NCRT and surgery increases the odds of pCR (14). Therefore, it is unclear whether it is the increased interval or the chemotherapy that enhances the CR rate.

The National Comprehensive Cancer Network (NCCN) classification of LARC encompasses a rather heterogeneous group of tumors and does not consider the extent of local invasion of the primary tumor. Currently, pelvic magnetic resonance imaging (MRI) is the most accurate test to evaluate patient risk and has been recommended for defining the clinical stage before NCRT. In the Mercury series study, MRI could predict long-term prognosis by detecting extramural vascular invasion (EMVI) status, T substage, and the nearest distance to the mesorectal fascia (MRF) (15). Accordingly, the European Society for Medical Oncology (ESMO) guidelines stratify new LARC risk groups and recommend corresponding treatments. For low-risk disease, defined as clinical T2-3b, middle and low, MRF-clear, and EMVI-negative, NCRT is recommended when good quality TME cannot be achieved or patients refuse surgery (16). Considering the good response of patients in this group to NCRT (17–19), non-surgical treatment strategies could be considered to preserve the anus. However, there are still some patients with minimal amount of residual tumor after NCRT. This retrospective study aimed to evaluate whether adding one or two cycles of capecitabine after NCRT without extending the interval between chemoradiation and surgery can benefit patients with low-risk middle-low LARC.

## Methods

### Patients

All consecutive patients treated at the Peking University Cancer Hospital between January 2015 and July 2019 were retrospectively identified. The inclusion criteria were as follows: (1) histologically confirmed rectal adenocarcinoma; (2) distal margin of the tumor located less than 10 cm from the anal verge; (3) defined as low-risk by primary MRI: clinical T2-3b, middle and low, MRF-clear, and EMVI-negative; (4) no systemic treatment before NCRT; (5) no evidence of distant metastasis at the initial diagnosis; and (6) age ≥18 years. Patients who did not achieve CR after NCRT and refused surgery were excluded from the study. Patients were divided into two groups based on whether consolidation chemotherapy was performed during the interval between NCRT and surgery. The study was approved by the review board of the Ethics Committee of Beijing Cancer Hospital, and all patients were informed of the benefits and risks of radiotherapy and signed informed consent forms before NCRT.

### MRI Assessment

A high-resolution, three-dimensional T2-weighted sequence MRI was mandatory before and after preoperative treatment (20). Either diagnostic or simulation MRI was available. The scanning layer thickness was 3–5 mm. Data regarding the length and thickness of the tumor, T3 substage, lymph node metastases, EMVI, and MRF status were evaluated. The criteria were based on the ESMO guidelines (16). Approximately 6-8 weeks after NCRT, MRI was performed again to assess the primary tumor response, regardless of whether the patient received consolidation chemotherapy.

### Neoadjuvant Regimens

Radiotherapy simulation was performed in the supine position on a thermoplastic film with contrast-enhanced computed tomography (CT). MRI simulation was recommended to help delineate rectal tumors. A full bladder and empty rectum were required to protect the intestine and ensure good repeatability. Target delineation was based on simulation CT. The details of the target contour and prescribed dose have been described previously (21). The regimens to the planning gross target volume (PGTV) and planning target volume (PTV) were 50.0-50.6 Gy and 41.8-45 Gy, respectively, in 22-25 fractions, five times per week for approximately 5 weeks. For concurrent chemotherapy, oral capecitabine was prescribed at a dose of 825 mg/m^2^ twice daily throughout the course of intensity modulated radiation therapy. For patients receiving consolidation chemotherapy, one or two cycles of capecitabine (1000 mg/m^2^ twice daily from days 1 to 14) were administered within 2 weeks of completing NCRT.

### Perioperative Evaluation

Following NCRT and consolidation chemotherapy, the tumor response and the development of distant metastases were comprehensively assessed by digital rectal examination, proctoscopy, pelvic MRI, and CT scan of the thorax and abdomen. The cCR was defined as the absence of viable tumor in the primary site and draining lymph nodes on MRI, negative biopsies from the scar, and normal (< 5 ng/mL) CEA levels (16). Patients who achieved cCR could select a non-operative strategy with rigorous and meticulous follow-up. For patients who did not achieve cCR, surgery was recommended after adequate radiological evaluation according to the TME criteria. The surgical procedures included transanal local excision, low anterior resection, abdominoperineal resection, and Hartmann surgery. The pathological stage was recorded according to the NCCN criteria (22). R0 resection was defined as a longitudinal margin and circumferential resection margin >1 mm (23).

### Follow-up

After completing the whole treatment, patients underwent follow-up evaluations every 3 months for the first 2 years, every 6 months for the 3 to 5 years, and then annually thereafter. Gastrointestinal tumor markers, symptoms, a physical examination, and chest and abdominal CT were required for all patients; digital rectal examination, pelvic MRI, and colonoscopy were required for patients diagnosed with cCR and pelvic CT or MRI was required for patients who underwent surgery.

### Outcome Measures

The primary outcome in this study was the CR rate. We defined the CR rate as the proportion of patients who achieved pCR as determined after surgery and the patients with sustained cCR under the “watch-and-wait” approach. Secondary outcomes included the ypT0-2 rate, ypN0 rate, toxicities, regrowth rate, distant metastasis-free survival (DMFS), and disease-free survival (DFS). Toxicities during neoadjuvant treatment were evaluated and recorded weekly in the outpatient department according to the Common Terminology Criteria for Adverse Events (version 3.0) criteria. For the patients who achieved cCR, the regrowth rate was recorded. DFS and DMFS were evaluated in patients who underwent surgery; DFS was defined from the date of surgery to any type of locoregional recurrence (LR), distant metastases (DM), or death for any reason; DMFS was defined from the date of surgery to any type of DM.

### Statistical Analysis

Data were collected and analyzed using the Statistical Package for the Social Sciences (IBM Corp. SPSS Statistics for Windows, version 22.0, Armonk, NY, USA). Statistical analyses included comparison of variables using the chi-square test for categorical variables and Student’s t-test for continuous variables. Survival curves were generated using the Kaplan-Meier method and compared using the log-rank test. Univariate and multivariate logistic regression analyses were used to evaluate the effects. Statistical significance was set at p < 0.05.

## Results

### Patient Characteristics

**Figure 1:**
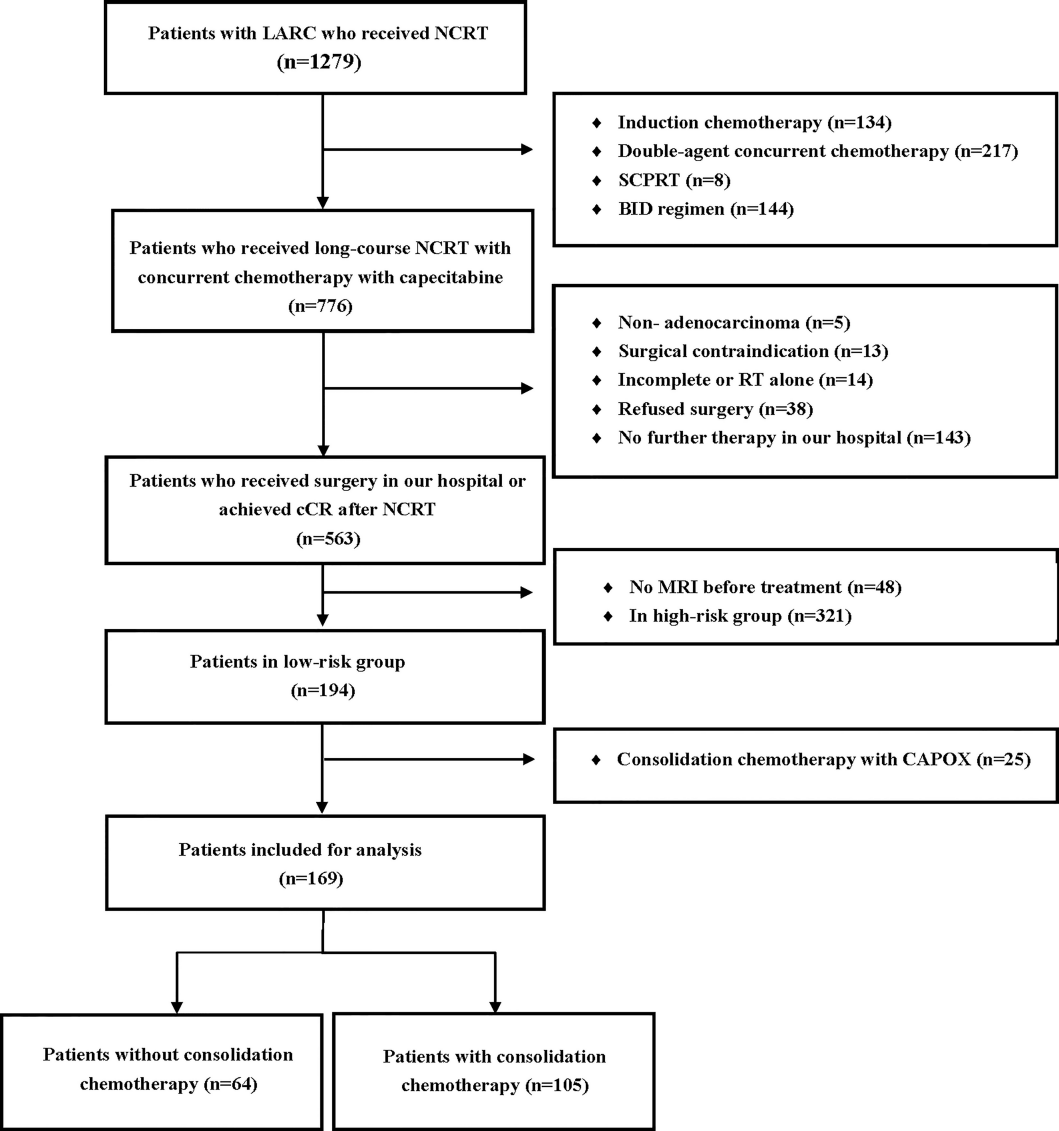
Flow diagram of the patient selection process based on the inclusion criteria and exclusion criteria. SCPRT, short-course preoperative radiotherapy; BID, radiotherapy delivered twice daily; CAPOX, capecitabine and oxaliplatin; LARC, locally advanced rectal cancer; NCRT, neoadjuvant chemoradiotherapy; cCR, clinical complete response; RT, radiotherapy.

A total of 169 patients were included in the study. A detailed flow diagram of the patient inclusion process is shown in **Figure 1**. One hundred and one (59.8%) patients were categorized as having low rectal cancer and 106 (62.7%) had normal CEA levels. One hundred and thirty-nine patients (82.2%) had stage cT3b disease. The median interval between the end of NCRT and surgery was 72 days (range, 41–256 days). One hundred and five patients received consolidation chemotherapy after NCRT and were placed in the consolidation chemotherapy group; 73 patients (69.5%) received one cycle of consolidation chemotherapy and 32 patients (30.5%) received two cycles. The remaining 64 patients did not receive consolidation chemotherapy and were assigned to the non-consolidation chemotherapy group. The baseline clinicopathological parameters of the two groups of patients are summarized in **Table 1**. Patients in the consolidation chemotherapy group were more likely to have positive lymph nodes than those in the non-consolidation chemotherapy group (p = 0.028), and other clinical parameters were well-balanced.

**Table 1:** The clinical parameters of the two groups.

	Consolidation chemotherapy group (n = 105)	Non-consolidation chemotherapy group (n = 64)	P value
Sex			0.139
Male	77 (73.3%)	40 (62.5%)	
Female	28 (26.7%)	24 (37.5%)	
Age (years)			0.281
Mean (SD)	59.2 (9.9)	60.9 (8.9)	
Primary tumor location			0.467
Middle	40 (38.1%)	28 (43.8%)	
Low	65 (61.9%)	36 (56.3%)	
Pathology			0.856
Well differentiated	5 (4.8%)	4 (6.3%)	
Moderately differentiated	84 (80.0%)	49 (76.6%)	
Poorly differentiated	4 (3.8%)	4 (6.3%)	
Others	12 (11.4%)	7 (10.9%)	
Clinical T stage			0.173
T2	7 (6.7%)	6 (9.4%)	
T3a	14 (13.3%)	3 (4.7%)	
T3b	84 (80.0%)	55 (85.9%)	
Clinical N stage			**0.028**
N0	9 (8.6%)	13 (20.3%)	
N+	96 (91.4%)	51 (79.7%)	
CEA (ng/mL)			0.545
<5	64 (60.9%)	42 (65.6%)	
≥5	29 (27.6%)	13 (20.3%)	
unidentified	12 (11.5%)	9 (14.1%)	
Tumor length (mm)			0.884
Mean (SD)	40.7 (12.5)	41.0 (10.0)	
Tumor thickness (mm)			0.247
Mean (SD)	14.1 (3.7)	14.8 (4.5)	
Interval time (days)			0.144
Mean (SD)	77.6 (29.5)	71.0 (23.2)	

SD, standard deviation; CEA, carcinoma embryonic antigen. Bold values means P < 0.05.

### Treatment and Pathological Outcomes

After neoadjuvant therapy, multidisciplinary assessments were performed. None of the patients had distant metastases during neoadjuvant therapy. Seventeen patients (10.1%) achieved cCR and received no surgery, and the remaining 152 patients (89.9%) underwent surgery. The median number of dissected lymph nodes was 7 (range, 0–24), and R0 resection was achieved in 100% of patients. Three (2.0%) patients received Hartman surgery, and 50 (32.9%) received APR, which cannot preserve anal sphincter function. Sixty-one of 92 patients (66.3%) in the consolidation chemotherapy group underwent sphincter-preserving surgery, and 38 of 64 patients (63.3%) in the non-consolidation chemotherapy group underwent sphincter-preserving surgery (P = 0.409). The surgery time, blood loss during surgery, and length of hospital stay after surgery were similar between the two groups.

In total, 64 patients (37.9%) were considered to have CR, 41 (39.0%) in the consolidation chemotherapy group and 23 (35.9%) in the non-consolidation chemotherapy group (p = 0.686). Among the patients who achieved CR in the consolidation chemotherapy group, there were 28 (28/73, 38.4%) patients who received one cycle consolidation and 13 (13/32, 40.6%) patients who received two cycles (p = 0.899). There were 13 (13/105, 12.4%) patients defined as having cCR and 28 (28/92, 30.4%) patients who achieved pCR in the consolidation chemotherapy group, and there were 4 (4/64, 6.3%) patients who achieved cCR and 19 (19/60, 31.7%) who achieved pCR in the non-consolidation chemotherapy group. The CR rates were 27.3% and 39.5% in patients with negative and positive lymph nodes, respectively (p = 0.272). Among patients who underwent surgery, 130 (85.5%) were diagnosed with ypN0 disease, 77 (83.7%) in the consolidation chemotherapy group and 53 (88.3%) in the non-consolidation chemotherapy group (p = 0.503). Ninety-eight patients (64.5%) were diagnosed with ypT0-2N0 disease, 60 (65.2%) in the consolidation chemotherapy group and 38 (63.3%) in the non-consolidation chemotherapy group (p = 0.812). There were no significant differences in the pathology results between the two groups. Details of the postoperative pathological stage and type of surgery are presented in **Table 2**.

**Table 2:** Details of pathological and surgical results in the two groups.

	Consolidation chemotherapy group (n = 105)	Non-consolidation chemotherapy group (n = 64)	P value
Clinical complete response	13 (12.4%)	4 (6.3%)	0.199
Distant metastasis	0 (0%)	0 (0%)	>0.99
Surgery (For total 152 patients)	N = 92	N = 60	
Surgical method			0.518
APR	30 (32.5%)	20 (33.3%)	
LAR	58 (63.1%)	38 (63.3%)	
Hartmann	1 (1.1%)	2 (3.3%)	
Local excision	3 (3.3%)	0 (0%)	
R0 resection	92 (100%)	60 (100%)	>0.99
Surgery time (minutes)			–
Median (range)	189 (34-540)	180 (69-420)	
Blood loss (mL)			–
Median (range)	50 (5-500)	50 (10-400)	
Length of hospital stay after surgery (days)			–
Median (range)	12 (7-33)	12 (6-27)	
Dissected lymph nodes (number)			–
Median (range)	8 (0-20)	7 (0-24)	
pT Stage			0.659
T0	29 (31.5%)	19(31.7%)	
T1	11 (12.0%)	5 (8.3%)	
T2	26 (28.3%)	14 (23.3%)	
T3	26 (28.3%)	22(36.7%)	
pN Stage			0.503
N0	77 (83.7%)	53 (88.3%)	
N1	14 (15.2%)	6 (10.0%)	
N2	1 (1.1%)	1 (1.7%)	
TRG Grade			0.819
0	29 (31.5%)	19 (31.7%)	
1	38 (41.3%)	21 (35.0%)	
2	25 (22.8%)	18 (30.0%)	
3	3 (3.3%)	1 (1.7%)	
unidentified	1 (1.7%)	1 (1.7%)	
pT0-2N0	60 (65.2%)	38 (63.3%)	0.812
pCR	28 (30.4%)	19 (31.7%)	0.872
cCR+pCR	41(39.0%)	23 (35.9%)	0.686

APR, abdominoperineal resection; LAR, low anterior resection; TRG, tumor regression grade; pCR, pathological complete response; cCR, clinical complete response.

The results from a univariate binary logistic regression analysis indicated that pretreatment CEA ≥5 ng/mL (OR, 0.187; 95% CI, 0.073-0.480; p < 0.001) and tumor thickness (OR, 0.917; 95% CI, 0.841-0.999; p = 0.048) were significantly associated with CR. Consolidation chemotherapy was not significantly associated with CR (OR, 0.876; 95% CI, 0.460-1.667; p = 0.686). Multivariate logistic regression analysis indicated that CEA ≥5 ng/mL (OR, 0.193; 95% CI, 0.075-0.498; p = 0.001) was associated with a lower CR rate. The results of the analysis are listed in **Table 3**.

**Table 3:** Univariate and multivariable analysis of factors affecting CR.

	Univariate analysis		Multivariate analysis	
	P value	OR (95% CI)	P value	OR (95% CI)
Age	0.904	1.002 (0.970-1.035)		
Sex (Male *vs* Female)	0.916	0.964 (0.492-1.889)		
Primary location (Middle *vs* Low)	0.126	0.603 (0.315-1.152)		
Pathology	0.656			
Well *vs* Moderately differentiated	0.363	0.474 (0.095-2.373)		
Poorly *vs* Moderately differentiated	0.996	0.996 (0.228-4.348)		
Others *vs* Moderately differentiated	0.416	1.494 (0.568-3.927)		
CEA (≥ 5ng/ml *vs* < 5ng/ml)	**0.000**	**0.187 (0.073-0.480)**	**0.001**	**0.193 (0.075-0.498)**
Clinical T-stage (T2 *vs* T3)	0.963	1.028 (0.321-3.289)		
Clinical N-stage (N0 *vs* N+)	0.276	0.575 (0.213-1.556)		
Tumor length	0.352	0.987 (0.960-1.015)		
Tumor thickness	**0.048**	**0.917 (0.841-0.999)**	0.159	0.934 (0.849-1.027)
Treatment group (non-consolidation *vs* consolidation chemotherapy)	0.686	0.876 (0.460-1.667)		

OR, odds ratio; CI, confidence interval; CEA, carcinoma embryonic antigen. Bold values means P < 0.05.

### Toxicities

**Figure 2:**
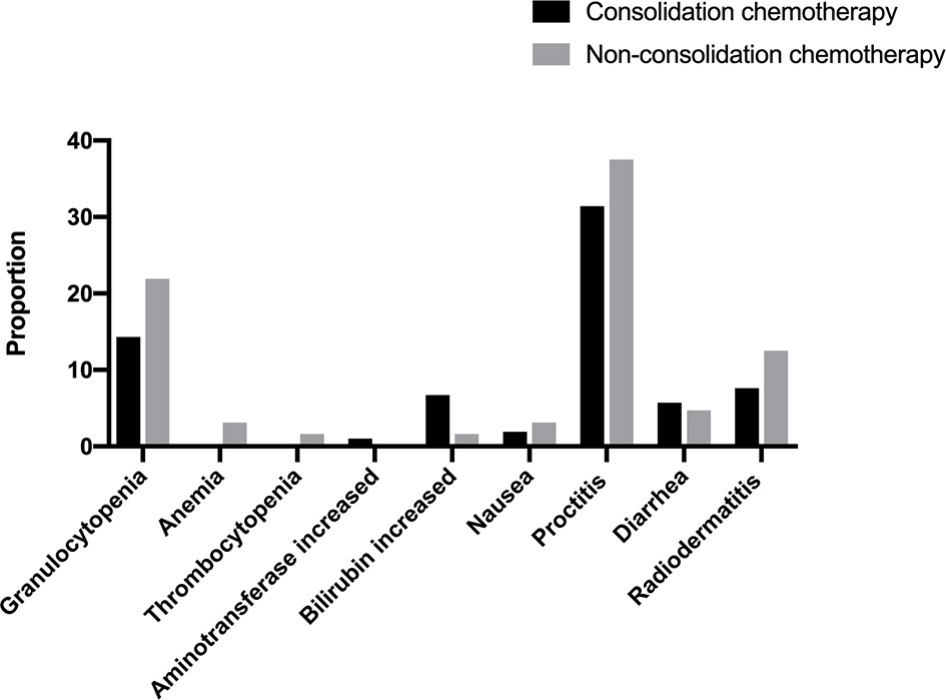
Toxicities during neoadjuvant treatment.

During neoadjuvant treatment, all patients completed the full-dose radiotherapy plan. Six patients experienced dose reductions of synchronous chemotherapy, 5 (7.8%) in the non-consolidation chemotherapy group and 1 (1.0%) in the consolidation chemotherapy group. In total, 90 patients (53.3%) had grade ≥2 acute toxicity, and the most common toxicities were proctitis (33.7%) and leukopenia (17.2%). Seven patients (4.2%) developed grade 3 acute toxicity. No grade 4 toxicities or toxicity-related deaths occurred during chemoradiotherapy. Grade ≥2 acute toxicity occurred in 34 (53.1%) patients in the non-consolidation chemotherapy group and 56 (53.3%) patients in the consolidation chemotherapy group (p = 0.979). **Figure 2** shows the details of toxicities during the neoadjuvant treatment.

### Long-Term Outcomes

**Figure 3:**
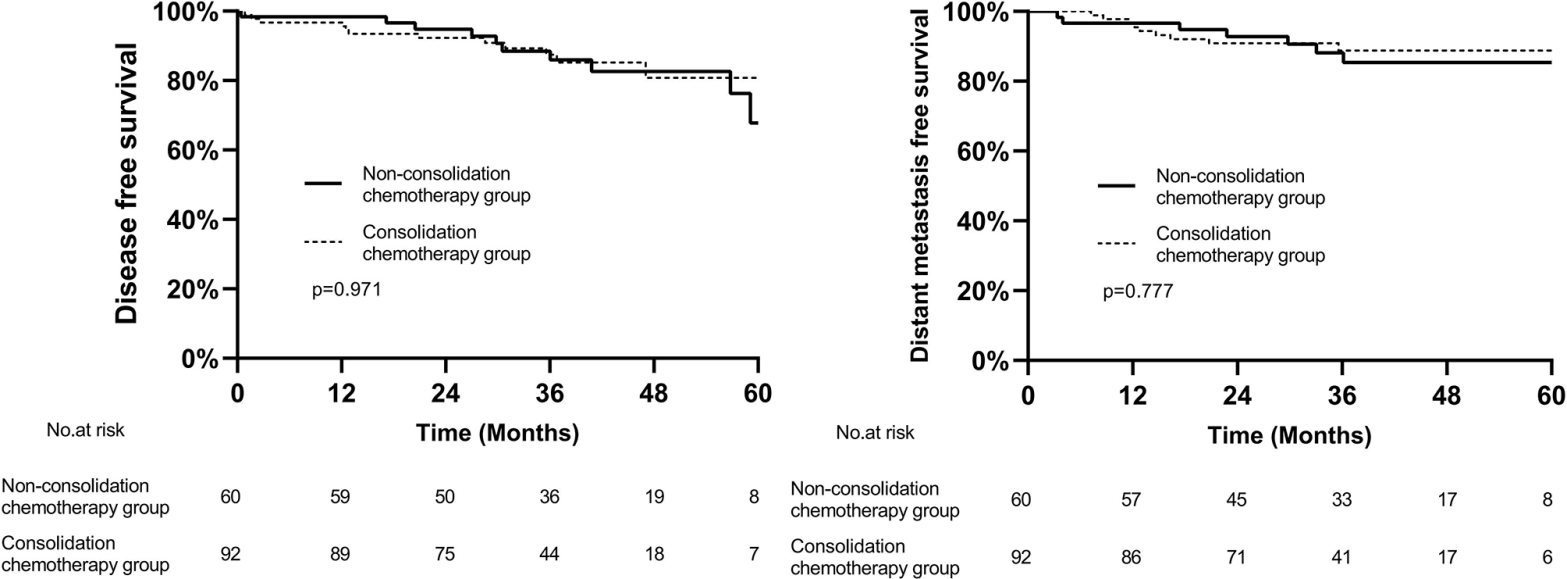
Kaplan–Meier curves of disease-free survival and distant metastasis free survival between two groups.

In the whole cohort, the median follow-up was 37.2 months (range, 0.4–71.2 months), 44.7 months (range, 16.2–71.3 months) in patients diagnosed with cCR and 36.6 months (range, 0.4–70.8 months) in patients who underwent surgery. Among the 17 patients who achieved cCR, 3 patients experienced regrowth in the rectum and underwent surgery, and all occurred within 2 years. Two of them developed distant metastasis, one in pelvic and one in lung and bone. In the patients who underwent surgery, the 3-year DFS and DMFS in consolidation chemotherapy group and non-consolidation chemotherapy group was 87.4% *vs* 85.9% (p=0.971) and 88.8% *vs* 85.4% (p=0.777), respectively (**Figure 3**).

## Discussion

To the best of our knowledge, this is the first study to explore the effectiveness of adding consolidation chemotherapy of one or two cycles of capecitabine after NCRT in patients with low-risk middle-low LARC. On the basis of not extending the interval between NCRT and surgery, one or two cycles of capecitabine consolidation chemotherapy did not improve the CR rate. The incidences of toxicities during neoadjuvant treatment were similar between the two groups.

MRI has become a necessary evaluation method before primary treatment for LARC and can be used to stratify patients. In the MERCURY study, patients who were defined as having low-risk disease by high-resolution MRI obtained good outcomes by TME alone; the local recurrence rate was 3% and the disease-free survival rate was 85% (15). However, the significant morbidity and mortality associated with surgery should not be overlooked. Approaches should be developed to ensure a curative effect while reducing treatment-related toxicities. Additionally, patients with low risk may be more likely to develop CR after NCRT alone, and approximately 50% of patients with cT2 disease and 25% with cT3 disease achieve pCR (17, 19). These patients should be considered for organ-preserving strategies. Habr-Gama et al. first compared the outcomes of patients who underwent a “watch-and-wait” approach and those who achieved pCR after surgery. The non-surgical group had slightly better OS (100% *vs*. 92%) and DFS (88% *vs*. 83%) at 5 years (8). Maas et al. reported that patients who underwent a nonoperative “watch-and-wait” approach had better functional outcomes than patients who underwent surgery (10). A number of multicenter studies on “watch-and-wait” have been conducted. The OnCoRe project (9) showed that patients managed by “watch-and-wait” had a significantly better 3-year colostomy-free survival than those who underwent surgical resection (74% *vs*. 47%, p < 0.001). In our study, the total CR rate was 37.9%. Seventeen patients (10.1%) achieved cCR, and these patients may have been able to retain organ preservation through neoadjuvant treatment alone.

Strategies to increase the chance of CR are still an important issue for LARC. Several studies have explored the effect of the interval between NCRT and surgery (14, 24, 25). A study based on the NCDB analyzed 17,255 LARC patients, and the results showed that an NCRT-surgery interval of more than 8 weeks was associated with a higher pCR rate (OR 1.12, 95% CI: 1.01-1.25) and a higher downstaging rate (OR 1.11, 95% CI: 1.02-1.25) (14). Rombouts et al. analyzed 217 patients with early-stage rectal cancer who received NCRT and found that the length of treatment interval did not affect outcomes in patients (26). The GRECCAR-6 trial evaluated 7-week and 11-week intervals, and the pCR rates were similar between the two groups (15.0% *vs*. 17.4%; p = 0.5983), but the quality of TME was better in the 11-week group (25). Huntington et al. examined 6397 LARC patients in the NCDB and found that an interval longer than 60 days was associated with a higher rate of positive surgical margins (p = 0.009) and a lower rate of sphincter-preserving surgery (p = 0.007) (27). Therefore, it would be best to improve the CR rate without prolonging the NCRT-surgery interval. However, in our study, although the addition of one or two cycles of consolidation chemotherapy did not extend the interval between NCRT and surgery, this strategy also did not increase the CR rate in patients with low-risk middle-low LARC.

The NCCN guidelines recommend TNT as a new neoadjuvant strategy. The Spanish GCR-3 study included 108 LARC patients to test the effect of four inductive cycles of capecitabine and oxaliplatin (CAPOX) followed by NCRT, and the median follow-up was 69.5 months. Although this new strategy did not improve the 5-year OS or DFS, induction chemotherapy improved treatment compliance (28). A multicenter phase II study by Garcia-Aguilar et al. showed that the pCR rate increased with an increase in the number of cycles of consolidation FOLFOX6 chemotherapy after NCRT (29). The CAO/ARO/AIO-12 study enrolled 306 patients with LARC and administered two modes of TNT; the total CR rates were 21% in the induction chemotherapy group and 28% in the consolidation chemotherapy group, and the toxicity and surgical complication rates were similar (13). However, these studies had varying results on the effect of induction or consolidation chemotherapy on the pCR rate, which may be related to the enrolled population, the consolidation chemotherapy regimen, and the number of cycles. In our study, the addition of one to two cycles of capecitabine did not affect the CR rate for low-risk LARC patients, indicating that the chemotherapy regimen may need to be optimized in order to increase pCR.

To increase the pCR rate of patients with LARC, several randomized phase III trials (30–32) have analyzed the effect of adding oxaliplatin to the regimen of concurrent chemotherapy with radiation. However, most showed negative results, and more treatment-related toxicities were observed. Although oxaliplatin-containing chemotherapy might increase the pCR rate, it also increased toxicities. Considering the convenience and safety of capecitabine, our study retrospectively analyzed one or two cycles of capecitabine as consolidation therapy. In 2013, Zhu et al. (33) published a phase II study using CAPOX with NCRT and consolidation chemotherapy with one cycle of capecitabine. Forty-two patients were enrolled, 38 underwent surgery and 6 (14.3%) achieved pCR; the toxicity of neoadjuvant therapy and the incidence of surgical complications were acceptable. Wang et al. evaluated the outcome of NCRT and four cycles of consolidation CAPOX for low-risk LARC patients, and the cCR rate was 42.1% (16/38) (34). In our study, the CR rate was 39.0% (41/105) in the consolidation chemotherapy group, suggesting that one to two cycles of capecitabine chemotherapy might be insufficient.

We must acknowledge that this research has some inherent limitations as a retrospective and small sample sized study. First, because of the lack of quality control, patients who refused surgery or who received surgery in other hospitals were not included in the analysis, and our results are not completely representative of the real-world situation. Second, the clinical lymph node staging was not well balanced in in the two groups, which might underestimate the consolidation chemotherapy effect in some ways. Additionally, a randomized controlled trial is required to confirm the results of this retrospective study.

In conclusion, one or two cycles of capecitabine as consolidation chemotherapy after NCRT did not improve the CR rate or increase the toxicities in low-risk patients with locally advanced middle-low rectal cancer. To improve patient outcomes, optimal scheduling of neoadjuvant therapy still needs to be explored.

## Data Availability Statement

The raw data supporting the conclusions of this article will be made available by the authors, without undue reservation.

## Ethics Statement

The studies involving human participants were reviewed and approved by review board of the Ethics Committee of Beijing Cancer Hospital. The patients/participants provided their written informed consent to participate in this study.

## Author Contributions

Conception and design of study: WW and YC. Acquisition of data/drafting the manuscript: XS and SL. Analysis and/or interpretation of data: YZ, JG, XZ, HW, JQ, and YL. All authors contributed to the article and approved the submitted version.

## Funding

1) Beijing Municipal Science & Technology Commission No. Z181100001718192; 2) Capital’s Funds for Health Improvement and Research No. 2020-2-1027, No. 2020-1-4021; 3) National Natural Science Foundation No. 82073333; 4) Beijing Natural Science Foundation No. 7182028; 5) Clinical Technology Innovation Project of Beijing Hospital Authority No. XMLX201842; 6) Science Foundation of Peking University Cancer Hospital No. 18-03.

## Conflict of Interest

The authors declare that the research was conducted in the absence of any commercial or financial relationships that could be construed as a potential conflict of interest.

## Publisher’s Note

All claims expressed in this article are solely those of the authors and do not necessarily represent those of their affiliated organizations, or those of the publisher, the editors and the reviewers. Any product that may be evaluated in this article, or claim that may be made by its manufacturer, is not guaranteed or endorsed by the publisher.
